# Low heart rate variability from 10-s electrocardiograms is associated with development of non-alcoholic fatty liver disease

**DOI:** 10.1038/s41598-022-05037-w

**Published:** 2022-01-20

**Authors:** In Young Choi, Yoosoo Chang, Geonggyu Kang, Hyun-Suk Jung, Hocheol Shin, Sarah H. Wild, Christopher D. Byrne, Seungho Ryu

**Affiliations:** 1 Total Healthcare Centre, Kangbuk Samsung Hospital, Sungkyunkwan University School of Medicine, Seoul, South Korea; 2 Centre for Cohort Studies, Total Healthcare Centre, Kangbuk Samsung Hospital, Sungkyunkwan University School of Medicine, Seoul, South Korea; 3 Department of Occupational and Environmental Medicine, Kangbuk Samsung Hospital, Sungkyunkwan University School of Medicine, Samsung Main Building B2, 250 Taepyung-ro 2ga, Jung-gu, Seoul, 04514 South Korea; 4 Department of Clinical Research Design and Evaluation, SAIHST, Sungkyunkwan University, Seoul, South Korea; 5 Department of Family Medicine, Kangbuk Samsung Hospital, Sungkyunkwan University School of Medicine, Seoul, South Korea; 6 Usher Institute University of Edinburgh, Edinburgh, UK; 7 Nutrition and Metabolism, Faculty of Medicine, University of Southampton, Southampton, UK; 8 National Institute for Health Research Southampton Biomedical Research Centre, University Hospital Southampton, Southampton, UK

**Keywords:** Cardiology, Gastroenterology, Medical research, Risk factors

## Abstract

Reduced heart rate variability (HRV) is reflective of autonomic imbalance. However, its impact on non-alcoholic fatty liver disease (NAFLD) is unknown. We investigated the association between 10-s HRV and incident NAFLD. A cohort of 154,286 Korean adults with no NAFLD at baseline were followed up. 10-s electrocardiograms were used to estimate two time-domain HRV, the standard deviation of normal-to-normal intervals (SDNN) and the root mean square of successive differences in RR intervals (RMSSD). Hepatic steatosis (HS) and liver fibrosis were assessed using ultrasonography and the fibrosis-4 index (FIB-4). A total of 27,279 incident HS (median follow up of 4.2 years) and 1250 incident HS plus high FIB-4 (median follow up of 4.2 years) cases were identified at follow-up. The multivariable adjusted hazard ratios (aHRs) (95% confidence intervals [CIs]) in a model with time-dependent variables for incident HS, comparing the lowest quintile to the highest and reference quintile of the RMSSD, was 1.43 (1.37–1.49), and the corresponding HR for incident HS plus intermediate/high FIB-4 was 1.70 (1.35–2.15). Similarly, SDNN was inversely associated with incident HS and HS plus intermediate/high FIB-4. The results were similar using the NAFLD fibrosis score. Autonomic imbalance assessed by HRV may help to identify individuals at a high risk of HS and its progression and warrant further studies.

## Introduction

Heart rate variability (HRV), defined by the variation in consecutive beat-to-beat intervals, reflects the constant interaction between the sympathetic and parasympathetic nervous systems and is widely used as a noninvasive tool for evaluating autonomic nervous system (ANS) imbalance^1,2^. A body of evidence suggests that reduced HRV is associated with cardiometabolic risk factors (obesity, hypertension, and diabetes), cardiovascular morbidity and mortality, and all-cause mortality^3–5^. Although 5‐min electrocardiographic (ECG) recordings are the standard measures for evaluating HRV^1^, recent studies have reported that HRV time-domain parameters derived from 10-s ECG recordings^6–9^ are also reliable measures for assessing HRV. Since the 10-s ECG is one of the most widely measured tests in clinical practice, the 10-s ECG-derived HRV can be used to evaluate the relationship between autonomic imbalance and health in large population-based studies.

Non-alcoholic fatty liver disease (NAFLD) is a leading cause of chronic liver disease worldwide, with an estimated global prevalence of approximately 25%^10,11^. NAFLD is associated with increased risks of mortality related to cardiometabolic disease, as well as increased liver-related morbidity and mortality^12^. As there are currently no licensed drugs available for the treatment of NAFLD, it is of considerable importance to identify modifiable risk factors and manage them to prevent this disease^10–12^.

The pathogenesis of NAFLD is not fully understood, although insulin resistance is implicated in both the development and progression of NAFLD^13^. The liver is innervated by both the sympathetic and parasympathetic nervous systems, both of which play an important role in the autonomic regulation of glucose and lipid metabolism^14,15^. Interestingly, several experimental studies have demonstrated that increased sympathetic nervous activity could induce NAFLD and influence its progression, even non-alcoholic steatohepatitis (NASH)-cirrhosis, whereas parasympathetic activation reduces progression^16^, suggesting its potential role in the autonomic imbalance in the pathogenesis of NAFLD. To date, no cohort studies have addressed whether low HRV, reflective of autonomic imbalance, is associated with the risk and progression of NAFLD.

Therefore, this study aimed to investigate the prospective association between HRV, based on 10-s ECG recordings, and the development of hepatic steatosis (HS) both with and without an intermediate/high probability of advanced fibrosis.

## Results

### Baseline characteristics

At baseline, the mean (SD) of SDNN and RMSSD were 36.8 ms (25.3) and 44.6 ms (37.7), respectively. Of the 154,286 participants with 611,478 person-years of follow-up (median follow-up of 4.2 years, interquartile range of 2.3–6.0 years), 27,279 cases of incident HS were identified (incidence rate 44.6 per 10^3^ person-years). The median frequency of visits was 3 (interquartile range, 2–5) and median interval between the baseline and 2nd visit was 1.8 years (interquartile range, 1.1–2.1). Incident HS was positively associated with age, male sex, obesity, current smoking, alcohol intake, hypertension, diabetes, medication for hyperlipidemia, unfavorable lipid profiles, and levels of liver enzymes, hsCRP, and HOMA-IR (Table 1).

**Table 1:** Baseline characteristics according to incident hepatic steatosis at follow-up (n = 160,150).

Characteristics	No hepatic steatosis	Hepatic steatosis	P-value
Number	127,007	27,279	< 0.001
Age (years)^a^	36.0 (6.5)	37.5 (6.5)	< 0.001
Male (%)	31.7	64.2	< 0.001
Obesity (%)^c^	7.8	27.3	< 0.001
Current smoker (%)	11.3	25.6	< 0.001
Alcohol consumption (%)^d^	22.5	35.9	< 0.001
HEPA (%)	14.2	15.6	< 0.001
High education level (%)^e^	85.5	86.0	0.042
Married (%)	70.5	68.4	< 0.001
Hypertension (%)	1.0	2.4	< 0.001
Diabetes (%)	0.2	0.5	< 0.001
Medication for hyperlipidemia (%)	0.4	0.9	< 0.001
Sleep duration (h)^a^	6.7 (1.2)	6.5 (1.1)	< 0.001
Depressive symptoms (%)^f^	12.6	10.0	< 0.001
Heart rate (beats/min)^a^	64.6 (8.7)	65.3 (8.8)	< 0.001
SDNN (ms)^b^	38.0 (26.2–54.8)	32.7 (22.4–47.6)	< 0.001
RMSSD (ms)^b^	37.7 (25.9–54.4)	32.2 (21.9–47.2)	< 0.001
Body mass index (kg/m^2^)	21.4 (2.5)	23.7 (2.5)	< 0.001
Systolic BP (mmHg)^a^	103.6 (11.1)	109.6 (11.5)	< 0.001
Diastolic BP (mmHg)^a^	66.0 (8.3)	69.8 (8.8)	< 0.001
Glucose (mg/dl)^a^	90.6 (7.8)	93.3 (8.7)	< 0.001
Total cholesterol (mg/dl)^a^	185.3 (30.9)	195.8 (32.2)	< 0.001
LDL (mg/dl)^a^	110.3 (28.4)	124.5 (29.5)	< 0.001
HDL (mg/dl)^a^	64.6 (14.6)	55.5 (12.9)	< 0.001
Triglyceride	71 (54–95)	97 (72–136)	< 0.001
Alanine aminotransferase (U/l)^b^	14 (11–18)	18 (14–25)	< 0.001
Aspartate aminotransferase (U/l)^b^	17 (15–20)	19 (16–23)	< 0.001
GGT (U/l)^b^	14 (11–20)	21 (15–32)	< 0.001
hsCRP (mg/l)^b^	0.3 (0.2–0.6)	0.5 (0.3–0.9)	< 0.001
HOMA-IR^b^	0.98 (0.67–1.39)	1.20 (0.82–1.70)	< 0.001
Total calorie intake (kcal/d)^b,g^	1457 (1098–1846)	1595 (1251–1990)	< 0.001

Data are expressed as the ^a^mean (standard deviation), ^b^median (interquartile range), or percentage.

^c^BMI ≥ 25 kg/m^2^; ^d^≥ 20 g/day; ^e^≥ college graduate; ^f^≥ 16 of CES-D score; ^g^among 111,388 participants with plausible estimated energy intake levels (within three standard deviations from the log-transformed mean energy intake).

*BP* blood pressure, *GGT* gamma-glutamyl transpeptidase, *HDL-C* high-density lipoprotein cholesterol, *HEPA* health-enhancing physically active, *LDL-C* low-density lipoprotein cholesterol, *RMSSD* the root mean square of successive differences in RR intervals, *SDNN* standard deviation of normal-to-normal intervals.

### Incident hepatic steatosis by heart rate variability

Both SDNN and RMSSD were inversely associated with the risk of incident HS. After adjustment for confounders, multivariable-adjusted HRs (95% CIs) for incident HS, comparing the first, second, third, and fourth quintiles with the highest quintile of the RMSSD, were only attenuated slightly: 1.31 (1.26–1.38), 1.20 (1.15–1.25), 1.13 (1.08–1.17), and 1.05 (1.01–1.10), respectively (*P for trend* < 0.001). In a model with time-dependent variables, HRs (95% CIs) for incident HS, comparing the first, second, third, and fourth quintiles with the highest quintile of the RMSSD were 1.43 (1.37–1.49), 1.25 (1.19–1.30), 1.18 (1.13–1.23), and 1.06 (1.02–1.11), respectively (*P for trend* < *0.001*). The HRs (95% CIs) for the four quintile with the highest quintile of SDNN (reference) in multivariable-adjusted model 2 were 1.22 (1.17–1.27), 1.14 (1.09–1.18), 1.08 (1.04–1.13), and 1.02 (0.98–1.06), respectively (*P for trend* < *0.001*). The corresponding HRs (95% CIs) for incident HS in time-dependent analysis, comparing the first, second, third, and fourth quintiles with the highest quintile of the SDNN were 1.30 (1.25–1.36), 1.19 (1.15–1.24), 1.10 (1.05–1.14), and 1.03 (0.99–1.08), respectively (*P for trend* < *0.001*). For time-dependent analyses, the updated status of HRV and other confounders were incorporated as time-varying covariates (Table 2).

**Table 2:** The incidence of hepatic steatosis by heart rate variability.

Quintiles of heart rate variability	PY	Incident cases	Incidence density (/10^3^ PY)	Age sex adjusted HR (95% CI)	Multivariable-adjusted HR^a^ (95% CI)		HR (95% CI)^b^ in a model with time-dependent variables
					Model 1	Model 2	
**SDNN quintiles**							
Q1	114,589	6739	58.8	1.39 (1.34–1.45)	1.32 (1.27–1.37)	1.22 (1.17–1.27)	1.30 (1.25–1.36)
Q2	122,431	5834	47.7	1.21 (1.17–1.26)	1.19 (1.15–1.24)	1.14 (1.09–1.18)	1.19 (1.15–1.24)
Q3	124,751	5311	42.6	1.12 (1.08–1.17)	1.12 (1.08–1.16)	1.08 (1.04–1.13)	1.10 (1.05–1.14)
Q4	124,845	4846	38.8	1.04 (1.00–1.09)	1.04 (1.00–1.08)	1.02 (0.98–1.06)	1.03 (0.99–1.08)
Q5	124,863	4549	36.4	1.00 (reference)	1.00 (reference)	1.00 (reference)	1.00 (reference)
*P for trend*				< 0.001	< 0.001	< 0.001	< 0.001
**RMSSD quintiles**							
Q1	113,273	7126	62.9	1.51 (1.45–1.57)	1.42 (1.37–1.48)	1.31 (1.26–1.38)	1.43 (1.37–1.49)
Q2	122,232	5992	49.0	1.29 (1.24–1.34)	1.26 (1.21–1.31)	1.20 (1.15–1.25)	1.25 (1.19–1.30)
Q3	125,323	5335	42.6	1.17 (1.12–1.22)	1.16 (1.12–1.21)	1.13 (1.08–1.17)	1.18 (1.13–1.23)
Q4	125,302	4718	37.7	1.07 (1.03–1.12)	1.08 (1.03–1.12)	1.05 (1.01–1.10)	1.06 (1.02–1.11)
Q5	125,348	4108	32.8	1.00 (reference)	1.00 (reference)	1.00 (reference)	1.00 (reference)
*P for trend*				< 0.001	< 0.001	< 0.001	< 0.001

^a^Estimated from parametric proportional hazard models. The multivariable model was adjusted for age, sex, centre, year of screening examination, smoking, alcohol consumption, physical activity, depressive symptoms, sleep duration, education level, total energy intake, and systolic blood pressure.

^b^Estimated from parametric proportional hazard models with quintiles of each heart rate variability (SDNN and RMSSD), smoking, alcohol consumption, physical activity, depressive symptoms, sleep duration, total energy intake, and systolic blood pressure as time-dependent categorical variables; and baseline age, sex, centre, year of screening exam, and education level as time-fixed variables.

*CI* confidence interval, *HR* hazard ratio, *PY* person-year, *RMSSD* root mean square of successive differences in RR intervals, *SDNN* standard deviation of normal-to-normal intervals.

### Incident hepatic steatosis with intermediate/high FIB-4 by heart rate variability

During a median follow-up of 4.2 years (interquartile range 2.3–6.0), 1250 cases of incident HS plus intermediate/high FIB-4, and 2834 cases of incident HS plus intermediate/high NFS were identified (incidence rate of 2.9 per 10^3^ person-years and 2.7 per 10^3^ person-years, respectively). Both SDNN and RMSSD were also significantly and inversely associated with the risk of incident HS plus intermediate/high FIB-4. Specifically, the multivariable-adjusted HR (95% CIs) in a model with time-dependent variables for incident HS plus intermediate/high FIB-4, comparing the first, second, third, and fourth quintile with the highest quintile of the RMSSD (reference), were 1.70 (1.35–2.15), 1.47 (1.16–1.88), 1.36 (1.06–1.75), and 1.30 (1.00–1.70), respectively. The lowest quintile of both SDNN and RMSSD also was significantly associated with an increased risk of incident HS plus intermediate/high NFS (Table 3).

**Table 3:** Hazard ratios (95% CI) for development of hepatic steatosis with intermediate-to-high probability of advanced fibrosis based on fibrosis-4 score and NAFLD fibrosis score by heart rate variability.

Quintiles of heart rate variability	For development of HS with intermediate-to-high probability of advanced fibrosis based on fibrosis-4 score			For development of HS with intermediate-to-high probability of advanced fibrosis based on NAFLD fibrosis score		
	Incidence density (/10^3^ PY)	Multivariable-adjusted HR^a^ (95% CI)	HR (95% CI)^b^ in a model with time-dependent variables	Incidence density (/10^3^ PY)	Multivariable-adjusted HR^a^ (95% CI)	HR (95% CI)^b^ in a model with time-dependent variables
**SDNN quintiles**						
Q1	2.9	1.16 (0.96–1.41)	1.51 (1.21–1.88)	4.7	1.41 (1.21–1.65)	1.56 (1.31–1.85)
Q2	2.2	1.22 (1.01–1.48)	1.45 (1.15–1.82)	3.0	1.20 (1.02–1.42)	1.36 (1.13–1.63)
Q3	1.7	1.08 (0.88–1.32)	1.40 (1.11–1.77)	2.4	1.09 (0.92–1.29)	1.26 (1.05–1.52)
Q4	1.4	1.02 (0.83–1.25)	1.36 (1.06–1.73)	2.0	1.02 (0.86–1.22)	1.14 (0.94–1.39)
Q5	1.2	1.00 (reference)	1.00 (reference)	1.8	1.00 (reference)	1.00 (reference)
*P for trend*		0.03	0.001		< 0.001	< 0.001
**RMSSD quintiles**						
Q1	3.2	1.34 (1.10–1.64)	1.70 (1.35–2.15)	4.9	1.50 (1.27–1.77)	1.67 (1.39–2.00)
Q2	2.2	1.28 (1.04–1.57)	1.47 (1.16–1.88)	3.0	1.26 (1.06–1.49)	1.37 (1.14–1.66)
Q3	1.8	1.23 (1.00–1.52)	1.36 (1.06–1.75)	2.6	1.25 (1.05–1.49)	1.25 (1.03–1.53)
Q4	1.3	1.00 (0.80–1.25)	1.30 (1.00–1.70)	1.9	1.08 (0.90–1.30)	1.21 (0.98–1.49)
Q5	1.1	1.00 (reference)	1.00 (reference)	1.5	1.00 (reference)	1.00 (reference)
*P for trend*		< 0.001	< 0.001		< 0.001	< 0.001

^a^Estimated from parametric proportional hazard models. The multivariable model was adjusted for age, sex, study centre, year of screening examination, smoking, alcohol consumption, physical activity, depressive symptoms, sleep duration, education level, total energy intake, and systolic blood pressure.

^b^Estimated from parametric proportional hazard models with quintiles of each heart rate variability (SDNN, RMSSD), smoking, alcohol consumption, physical activity, depressive symptoms, sleep duration, total energy intake and systolic blood pressure as time-dependent categorical variables; and baseline age, sex, centre, year of screening exam, and education level as time-fixed variables.

*CI* confidence interval, *HR* hazard ratio, *HS* hepatic steatosis, *PY* person-year, *RMSSD* root mean square of successive differences in RR intervals, *SDNN* standard deviation of normal-to-normal intervals.

### Sensitivity and mediation analysis

In a sensitivity analysis based on NFS, low values of both SDNN and RMSSD were significantly associated with an increased risk of incident HS plus intermediate/high NFS in fixed and time-dependent models. After further adjustment for BMI or HOMA-IR, the association between RMSSD and incident HS plus intermediate/high FIB-4 was attenuated and no longer significant; after adjustment for hsCRP, the association was attenuated but remained significant. For analyses based on NFS instead of FIB-4, the BMI was not adjusted because it is a component of the NFS score. Further adjustment for HOMA-IR or hsCRP attenuated the association between HRV and HS plus intermediate/high NFS, but the associations remained statistically significant (Supplementary Table 1). All these associations between low HRV measured by both SDNN and RMSSD and a increased risk of HS or HS plus intermediate/high fibrosis markers based on FIB-4 were similarly observed in sub-groups of non-obese individuals (Supplementary Table 2).

When NAFLD was used as endpoint which restricted to only HS without secondary causes (use of steatogenic medication, significant alcohol consumption, and B or C viral seropositivity) throughout the follow-up period was used as an endpoint, the associations between low HRV, SDNN, RMSSD, and incident NAFLD were similarly observed (Supplementary Table 3). Similarly, low RMSSD was inversely and significantly associated with incident NAFLD with an intermediate-to-high probability of advanced fibrosis, while low SDNN tended to show an elevated risk of incident NAFLD with an intermediate/high FIB-4 score; however, this association was not significant in the multivariable-adjusted model (P for trend = 0.087) (Supplementary Table 4).

We additionally performed a sensitivity analysis using Hepatic Steatosis Index (HSI) of > 36 as an endpoint which has been validated for detection of HS in a Korean population^17^. In the sensitivity analysis using HSI > 36 as an endpoint, a low SDNN and a RMSSD were independently associated with an increased incidence of suspected HS (Supplementary Table 5).

Additionally, the associations between potential confounders with HRV and HS are presented in Supplementary Tables 6–8.

## Discussion

In this large-scale cohort study of apparently healthy adults, low HRV, measured by the SDNN and RMSSD, which reflect global autonomic regulation and parasympathetic activity of the heart, respectively, were associated with increased risk of both incident HS and HS plus intermediate/high fibrosis markers based on FIB-4 or NFS. This association was similarly observed when changes in HRV and confounders during follow-up were treated as time-varying covariates and was independent of a wide range of potential confounders, including sleep duration and depressive symptoms, which influence HRV^18,19^. Similar findings were observed in analyses among non-obese individuals to address potential residual confounding by obesity, all of which are strong risk factors for NAFLD and its progression^20,21^. Our findings indicate that ANS function estimated by HRV may play an independent role in NAFLD risk and severity.

To the best of our knowledge, no previous cohort study has investigated the prospective association between HRV and NAFLD development. Our novel findings support and extend the results of previous cross-sectional studies^22–25^. One previous cross-sectional study of 497 subjects by Liu et al. (with a measure of 5 min HRV) showed a significant association between autonomic alteration (decreased SDNN and RMSSD) and increased prevalence of NAFLD, which was independent of traditional CVD risk factors, and HOMA-IR25. Another cross-sectional study of 96 sedentary individuals by Houghton et al. found that increased levels of fat in the liver were significantly associated with impaired cardiac and autonomic function, including detailed HRV parameters, when compared with individuals without fatty liver^23^. However, previous studies were limited by temporal ambiguity due to the cross-sectional study designs, relatively small sample sizes^22–25^, and the lack of adjustment for important confounders, including comorbidities^23,24^.

In our cohort study of 160,150 adults with no evidence of NAFLD at baseline, low HRV, measured by both SDNN and RMSSD, was significantly and consistently associated with an increased risk of developing both HS and HS plus intermediate/high fibrosis based on either FIB-4 or NFS. On the other hand, whereas low HRV measured by both SDNN and RMSSD was significantly associated with incident NAFLD and NAFLD plus intermediate/high NFS, only low HRV measured by both RMSSD was significantly associated with incident NAFLD plus intermediate/high FIB-4 in multivariable proportional hazard model. There are several possible explanations for this observation. First, SDNN reflects the overall dysfunction of the ANS, while the RMSSD is more influenced by PNS than SDNN, and is reflective of PNS activity. PNS alteration might precede SNS alteration^26,27^, and the RMSSD is considered a sensitive and reliable marker for parasympathetic activity^1,2^. Second, previous validation studies suggest that even a single 10-s (standard ECG) recording yields valid RMSSD measurements, while reliable measures for SDNN require a longer duration of at least 30 s or multiple 10-s ECGs^6,8,9^. Thus, in our study, HRV estimation based on a single 10-s ECG recording might explain the consistent finding based on the RMSSD; the RMSDD may be a more reliable HRV measure than SDNN in the setting using a single 10-s ECG recording, but also reflects the relative importance of PNS activity in NAFLD risk and its progression.

The mechanisms underlying the association between HRV and NAFLD are not yet fully understood, but there is growing evidence to suggest that autonomic dysfunction affects the risk of NAFLD and disease progression. Preclinical studies have demonstrated that an increase in SNS activity and adrenergic signaling causes HS and NASH-cirrhosis, while an increase in PNS activity inhibits the process from HS to NASH-cirrhosis^16^. Moreover, selective hepatic sympathetic denervation in mice led to almost complete resolution of HS in 1 week^28^, while hepatic vagotomy was found to exacerbate HS and hepatic inflammation in a mouse model^16,29^. In a human study of 34 individuals with biopsy-proven NAFLD and 34 matched controls, objective measures of ANS dysfunction based on 24-h BP and head tilt testing were prevalent in the group with NAFLD^30^. Furthermore, ANS dysfunction may also influence insulin resistance and inflammation, both of which are involved in the key pathogenesis of NAFLD^31–33^. Several studies have found that lowered HRV is associated with increased levels of inflammatory markers and HOMA‐IR^31–33^. Furthermore, an altered balance of the PNS and SNS activity, mainly explained by attenuated PNS activity, might contribute to the development of insulin resistance^34^. Additionally, sympathetic over activity might have an important role in metabolic alterations including central obesity and insulin resistance^35^. Intriguingly, HRV appears to improve with weight loss or adoption of a healthy lifestyle, including regular exercise, diet choice, and adequate sleep^18,19^. As the balance of ANS is a modifiable risk factor^18,19,36^, improving ANS function might help to reduce the development of NAFLD and its progression.

ANS dysfunction has been reported to increase the risk of obesity, and, at the same time, obesity may also influence autonomic imbalance, indicating that the two conditions affect each other^37^. Given that obesity is a strong risk factor for NAFLD^21^, we evaluated whether obesity and obesity-associated derangements, including insulin resistance and chronic inflammation, mediate the association between HRV and incident NAFLD by further adjusting for BMI, HOMA-IR, and hsCRP. The association between low HRV measured by SDNN and RMSSD and incident HS was attenuated but remained statistically significant, and the association with incident HS plus intermediate/high FIB-4 was more attenuated; however, longer follow-up studies may be required to validate this finding. Another possible explanation is that ANS dysfunction might be involved in the early stage of NAFLD, independent of obesity and its related metabolic abnormalities, whereas fibrosis progression appears to be more complex and mediated by obesity and its consequences related to ANS dysfunction.

This study has several limitations that should be discussed. First, because we used HRV based on 10-s ECG, more detailed information on high frequency, low frequency, or their ratio, all of which are also known to reflect the balance between sympathetic and parasympathetic activity, were unavailable. Second, HS and liver fibrosis were determined by liver ultrasonography and noninvasive fibrosis scores in the absence of liver biopsy. FIB-4 and NFS are the most commonly used and validated indices for excluding or identifying advanced liver fibrosis^38^. However, these indices can reliably exclude advanced fibrosis and not all cases classified as having an intermediate-to-high probability of advanced fibrosis may have advanced fibrosis^39^; thus, future studies using more sophisticated measures are required to assess the association between HRV and severe NAFLD. Lastly, the majority of participants in this cohort were relatively young, healthy, and well-educated Korean workers and their spouses, possibly limiting the generalizability of our findings to other populations and different age groups.

In conclusion, low HRV measured by 10-s ECG was associated with an increased risk of incident HS and HS plus intermediate/high fibrosis markers, independent of other existing risk factors. This finding supports that ANS dysfunction, especially decreased RMSSD, which is reflective of decreased parasympathetic activity, is involved in the pathophysiology of HS and its progression to fibrosis. Further studies are needed to clarify the exact role of ANS dysfunction in the pathogenesis of NAFLD and its progression, and to confirm whether approaches that improve HRV can help prevent NAFLD and its consequences.

## Methods

### Study population

**Figure 1:**
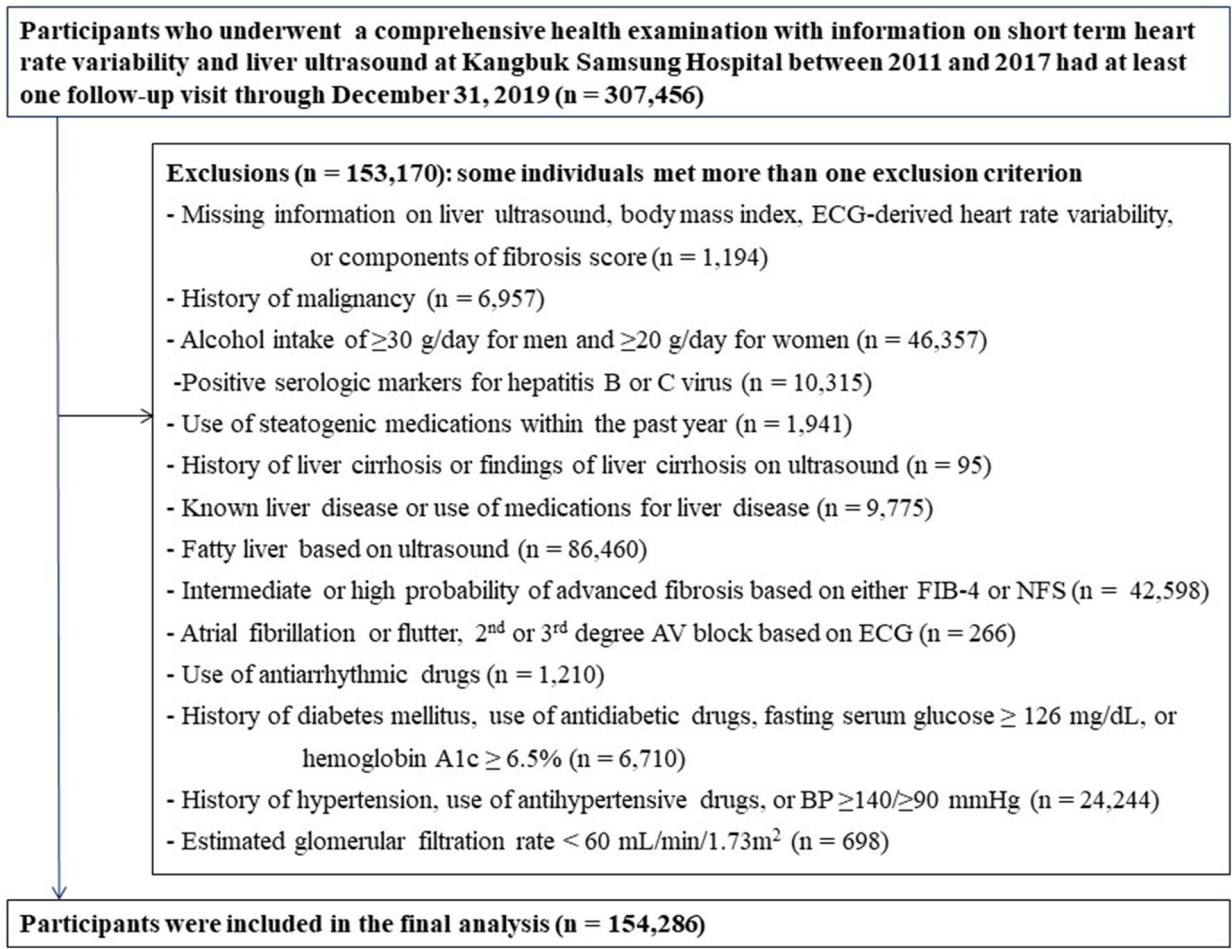
Flowchart of study participants.

This cohort study is part of the Kangbuk Samsung Health Study including Korean adults who underwent a comprehensive health screening examination at the Kangbuk Samsung Hospital Total Healthcare Centres located in Seoul and Suwon, South Korea^40^. To investigate the association between HRV and incident HS with/without intermediate/high fibrosis score, study participants were restricted to those who received a health examination from 2011 (the year from which 10-s ECG recordings were available) to 2017 and had at least one follow-up visit by December 31, 2019 (n = 307,456). A total of 153,170 participants were excluded based on the criteria listed in Fig. 1. The final analytic sample included 154,286 participants. All procedures involved in this study were in accordance with the 1964 Helsinki declaration and approved by the Institutional Review Board of Kangbuk Samsung Hospital (IRB 2021-01-033), which waived the requirement for informed consent since we performed analysis using a de-identified dataset that was routinely collected during the health screening process.

### Data collection

Health screening examinations included liver ultrasound, ECG, and blood laboratory and anthropometric measurements at both baseline and follow-up visits. A standardized, self-administered questionnaire was used to collect information on demographic characteristics, medical history, depressive symptoms and lifestyle behaviors (see further details in the supporting information).

Abdominal ultrasound was performed by experienced radiologists who diagnosed HS based on standard criteria, including the presence of a diffuse increase in fine echoes in the liver parenchyma compared with the kidney or spleen parenchyma, deep beam attenuation, and bright vessel walls^41^. The inter-observer and intra-observer reliability values for HS diagnoses were substantial (kappa statistic = 0.74) and excellent (kappa statistic = 0.94), respectively^40^.

The severity of NAFLD was assessed based on two noninvasive indices of liver fibrosis: the Fibrosis 4 score (FIB-4) and the NAFLD fibrosis score (NFS), which are adopted and recommended by most guidelines^38,42^. Subjects were classified as having low, intermediate, or high risk for advanced liver fibrosis for each score based on the following cutoff: FIB-4 (< 1.30, 1.30–2.66, and ≥ 2.67, respectively)^38^ and NFS (< − 1.455, − 1.455 to 0.676, and > 0.676, respectively)^42^. As the number of incidents with high probability of advanced fibrosis was not sufficient to examine a separate category, the combined outcome of intermediate or high fibrosis score was used as an endpoint.

Standard 12-lead, 10-s ECGs were performed as a routine part of the health screening process in all subjects on the same day as the other examinations were performed. The two time-domain HRV parameters, the standard deviation of normal-to-normal RR intervals (SDNN), and the root mean square of successive differences (RMSSD) were calculated based on the 10-s ECG recordings. The SDNN is affected by both sympathetic and parasympathetic nervous system (PNS) activity, while the RMSSD is the primary time-domain known to be more influenced by the PNS than the SDNN^2^. Low values of either SDNN or RMSSD are correlated with an increased risk of both cardiovascular morbidity and mortality^2–5^.

Physical activity levels were determined using the validated Korean version of the international Physical Activity Questionnaire (IPAQ) Short Form^43,44^. Health-enhancing physical activity (HEPA) was defined as physical activity that meets one of two following criteria: (1) vigorous activity on three or more days per week with ≥ 1500 of accumulated metabolic equivalent (MET)-min/week (1 MET is energy expenditure at rest); or (2) 7 days of any combination of walking, moderate intensity, or vigorous intensity activities achieving at least 3000 MET min/week^44^.

The Pittsburgh Sleep Quality Index (PSQI) was used to assess sleep quality and sleep duration over the month prior to study initiation^45^. Sleep duration was categorized as ≤ 5, 6, 7, 8, and ≥ 9 h. Depressive symptoms were assessed using the Korean version of the Centre for Epidemiologic Studies Depression (CES-D) Scale, and were categorized as CES-D scores < 16 and ≥ 16^46,47^.

The sitting blood pressure (BP) and anthropometric parameters were measured by trained nurses. Obesity was defined as a body mass index (BMI) ≥ 25 kg/m^2^, the proposed cutoff for obesity diagnosis in Asians^48^. Hypertension was defined as a systolic BP ≥ 140 mmHg, diastolic BP ≥ 90 mmHg, or current use of antihypertensive medication.

Blood specimens were obtained after at least 10 h of fasting. Blood measurements included lipid profiles, liver enzymes, glucose, hemoglobin A1c, insulin, high-sensitivity C-reactive protein (hsCRP), and platelet count. The homeostatic model assessment of insulin resistance (HOMA-IR) index was calculated as follows: fasting blood insulin (mU/mL) × fasting blood glucose (mmol/L)/22.5. Diabetes mellitus was defined as a fasting serum glucose level ≥ 126 mg/dL, hemoglobin A1c ≥ 6.5%, or current use of medication for the treatment of diabetes.

### Assessment of electrocardiogram

ECG was performed as a routine part of the health screening process in all subjects on the same day as the other examinations were performed. All ECGs were performed in a silent and relaxing private space in the absence of external stimuli, but the testing order was not specified. Thus, in some participants, the ECG was conducted after blood sampling, which may not have occurred due to exposure or outcome status; thus, this type of non-differential measurement error would have underestimated the true association between HRV and outcomes. Standard 12-lead ECGs were obtained at a paper speed of 25 mm/s and an amplification of 1 mV/cm with an ECG recorder (CARDIMAX FX-7542; Fukuda Denishi Co., Ltd., Tokyo, Japan). All ECGs were initially inspected for technical errors and quality by trained ECG technicians and then interpreted by three experienced cardiologists who were blinded to the aim of this study. All ECG data were digitalized and analyzed using an automated ECG analysis system (PI-19E; Fukuda Denishi Co., Ltd., Tokyo, Japan), which automatically detects the QRS complex and calculates the RR intervals.

### Statistical analysis

The baseline characteristics of the study participants are presented as percentages, means (standard deviations), and medians (interquartile ranges) according to incident HS. The SDNN and RMSSD were categorized into quintiles based on their own distributions because there is no clear established cutoff value of either measurement to define autonomic dysfunction. The highest quintile was set to the reference group given that low HRV is known to be associated with cardiometabolic disease^2–5^. The primary endpoints were: (a) development of incident HS (regardless of fibrosis score), and (b) the development of incident HS plus intermediate/high probability of liver fibrosis at follow-up. Incident HS and incident HS plus an intermediate or high probability of liver advanced fibrosis based on FIB-4 or NFS were treated as separate endpoints in each model.

The risk of incident HS and incident HS plus an intermediate/high probability of liver fibrosis were separately evaluated according to each HRV index. The hazard ratios (HRs) and 95% CIs were calculated using the parametric proportional hazards model. The models were initially adjusted for age and sex, and were then further adjusted for study centre (Seoul, Suwon), year of screening exam, smoking (never, past, current, or unknown), alcohol intake (none, < 10 or ≥ 10 g/day, or unknown), physical activity (inactive, minimally active, health-enhancing physical activity, or unknown), CES-D (< 16, ≥ 16, or unknown), sleep duration (≤ 5, 6, 7, 8, ≥ 9 h, or unknown), education level (< community college graduate, ≥ community college graduate, or unknown), total energy intake (quintiles or unknown), and systolic BP. Furthermore, to evaluate the impact of changes in HRV and covariates over follow-up, we conducted additional analyses by introducing HRV and other covariates as time-varying covariates in the models. To explore whether BMI, HOMA-IR, or hsCRP mediates the association between HRV and the primary endpoints, we included these variables in a multivariable-adjusted model (see further details in the supporting information). We assessed the proportional hazards assumption by examining graphs of estimated log (− log(survival)) versus the log of the survival time graph; no violation of the assumption was found.

The event detection date was defined as the earliest date of HS or HS identification with an intermediate or high probability of liver fibrosis. The person-years were calculated as the sum of the follow-up duration from baseline to the event detection date, or until the final examination (before December 31, 2019), whichever came first. The incidence rates were calculated as the number of incident cases divided by the person-years of follow-up. Because the primary endpoints would have occurred at an unknown time point between the event detection date and the previous screening visit, a parametric proportional hazards model was used to account for this type of interval censoring. In these models, the baseline hazard function was parameterized with natural cubic splines of log time with 3 internal knots at the 25th, 50th, and 75th percentiles (Stata package stpm)^49^.

To explore whether BMI, HOMA-IR, or hsCRP mediates the association between HRV and the primary endpoints, we included these variables in a multivariable-adjusted model. The following criteria for potential mediators were used: (1) The predictor of interest (HRV) was significantly related to the mediator (BMI, HOMA-IR, or hsCRP); (2) the mediator (BMI, HOMA-IR, or hsCRP) was significantly related to the outcome (HS or HS plus fibrosis); and (3) the addition of the mediator (BMI, HOMA-IR, or hsCRP) to the model both attenuated the coefficient of the predictor and had a statistically significant mediation effect.

We then evaluated whether or not the associations between HRV and the risk of HS and incident HS combined with an intermediate/high probability of liver fibrosis existed in individuals without obesity. The interactions between subgroup characteristics and HRV categories on the risk of HS and the intermediate/high probability of advanced liver fibrosis were tested using likelihood ratio tests, which compare models with and without multiplicative interaction terms. Statistical analyses were performed using STATA version 16.0 (StataCorp LP, College Station, TX, USA). All reported P-values were two-tailed, and differences were considered statistically significant at P < 0.05.

### Ethics approval and consent to participate

This study was approved by the Institutional Review Board of Kangbuk Samsung Hospital (IRB 2021-01-033), which waived the requirement for informed consent since we performed analysis using a de-identified dataset that was routinely collected during the health screening process.

### Consent for publication

The authors agree to the publication of this study.

## Supplementary Information

Supplementary Tables.

## Data Availability

Data are available from the Kangbuk Samsung Health Study whose authors may be contacted through the corresponding author. Unfortunately, the data are not available to be shared publicly as we do not have IRB permission for distributing the data.

## Abbreviations

ALT: Alanine aminotransferase
ANS: Autonomic nervous system
AST: Aspartate aminotransferase
BMI: Body mass index
BP: Blood pressure
CES-D: Centre for Epidemiologic Studies Depression
CI: Confidence interval
hs-CRP: High-sensitivity C-reactive protein
CVD: Cardiovascular disease
ECG: Electrocardiography
GGT: Gamma-glutamyl transferase
HDL-C: High-density lipoprotein cholesterol
FIB-4: Fibrosis-4 scores
HEPA: Health-enhancing physical activity
HOMA-IR: Homeostatic model assessment–insulin resistance
HR: Hazard ratio
HRV: Heart rate variability
HS: Hepatic steatosis
HSI: Hepatic Steatosis Index
LDL-C: Low-density lipoprotein cholesterol
NAFLD: Non-alcoholic fatty liver disease
NFS: NAFLD fibrosis score
PNS: Parasympathetic nervous system
PSQI: Pittsburgh Sleep Quality Index
RMSSD: Root mean square of successive differences in RR intervals
SDNN: Standard deviation of normal-to-normal intervals
SNS: Sympathetic nervous system
