# Author Correction: Low heart rate variability from 10-s electrocardiograms is associated with development of non-alcoholic fatty liver disease

**DOI:** 10.1038/s41598-022-25977-7

**Published:** 2022-12-09

**Authors:** In Young Choi, Yoosoo Chang, Geonggyu Kang, Hyun-Suk Jung, Hocheol Shin, Sarah H. Wild, Christopher D. Byrne, Seungho Ryu

**Affiliations:** 1 Total Healthcare Centre, Kangbuk Samsung Hospital, Sungkyunkwan University School of Medicine, Seoul, South Korea; 2 Centre for Cohort Studies, Total Healthcare Centre, Kangbuk Samsung Hospital, Sungkyunkwan University School of Medicine, Seoul, South Korea; 3 Department of Occupational and Environmental Medicine, Kangbuk Samsung Hospital, Sungkyunkwan University School of Medicine, Samsung Main Building B2, 250 Taepyung-ro 2ga, Jung-gu, Seoul, 04514 South Korea; 4 Department of Clinical Research Design and Evaluation, SAIHST, Sungkyunkwan University, Seoul, South Korea; 5 Department of Family Medicine, Kangbuk Samsung Hospital, Sungkyunkwan University School of Medicine, Seoul, South Korea; 6 Usher Institute University of Edinburgh, Edinburgh, UK; 7 Nutrition and Metabolism, Faculty of Medicine, University of Southampton, Southampton, UK; 8 National Institute for Health Research Southampton Biomedical Research Centre, University Hospital Southampton, Southampton, UK

Correction to: *Scientific Reports* https://doi.org/10.1038/s41598-022-05037-w, published online 20 January 2022

The Funding section in the original version of this Article was incomplete.

“This study was supported by SKKU Excellence in Research Award Research Fund, Sungkyunkwan University, 2020 and by the National Research Foundation of Korea (NRF) funded by the Ministry of Science, ICT and Future Planning (NRF-2017R1A2B2008401). CDB is supported in part by the Southampton NIHR Biomedical Research Centre (IS-BRC-20004), UK.”

now reads:

“This study was supported by SKKU Excellence in Research Award Research Fund, Sungkyunkwan University, 2020 and by the National Research Foundation of Korea (NRF) funded by the Ministry of Science, ICT and Future Planning (NRF-2017R1A2B2008401). CDB is supported in part by the Southampton NIHR Biomedical Research Centre (IS-BRC-20004), UK. This research was also supported by CDM-based precision medical data integration platform technology development program through the Korea Evaluation Institute of Industrial Technology funded by the Ministry of Trade, Industrial and Energy (No. 20005037)."

The original Article has been corrected.

