# Supplementary material for: Low heart rate variability from 10-s electrocardiograms is associated with development of non-alcoholic fatty liver disease
Source: Sci Rep. 2022 Jan 20;12:1062. doi: 10.1038/s41598-022-05037-w (PMC8776891; doi:10.1038/s41598-022-05037-w)
Supplement: Supplementary file 1 — Supplementary Tables. [file 41598_2022_5037_MOESM1_ESM.docx]

**Supporting information**

**Low heart rate variability from ten-second electrocardiograms is asscoicated with development of non-alcoholic fatty liver disease**

In Young Choi, Yoosoo Chang, Geonggyu Kang, Hyun-Suk Jung, Hocheol Shin, Sarah H Wild, Christopher D Byrne, Seungho Ryu

**Table of Contents**

**Supplementary Table 1.** Mediation analysis of the association between heart rate variability and the development of hepatic steatosis (HS) or HS with intermediate-to-high probability of advanced fibrosis fibrosis-4 score and NAFLD fibrosis score

**Supplementary Table 2.** Hazard ratios (95% CI) for the development of hepatic steatosis (HS) or HS with intermediate-to-high probability of advanced fibrosis measured by FIB-4 score, according to heart rate variability, among participants without obesity

**Supplementary Table 3.** Incidence of non-alcoholic fatty liver disease (NAFLD) by heart rate variability

**Supplementary Table 4.** Hazard ratios (95% CI) for development of non-alcoholic fatty liver disease (NAFLD) with intermediate-to-high probability of advanced fibrosis based on fibrosis-4 score and NAFLD fibrosis score by heart rate variability

**Supplementary Table 5.** Incidence of high Hepatic Steatosis Index (>36) heart rate variability

**Supplementary Table 6.** Hazard ratio (95% CI) of HR (95% CI) of hepatic steatosis by other covariates

**Supplementary Table 7.** Baseline characteristics of study participants by SDNN quintiles

**Supplementary Table 8.** Baseline characteristics of study participants by RMSSD quintiles

**Supplementary Table 1.** Mediation analysis of the association between heart rate variability and the development of hepatic steatosis (HS) or HS with intermediate-to-high probability of advanced fibrosis fibrosis-4 score and NAFLD fibrosis score

| **Quintiles of heart rate variability** | Multivariable-adjusted HR^a^ (95% CI) | | | |
| --- | --- | --- | --- | --- |
|  | Model 1  (including BMI) | Model 2  (including HOMA–IR) | Model 3  (including hsCRP) | Model 4  (including BMI, HOMA–IR, and hsCRP) |
| **Incident HS** |  |  |  |  |
| RMSSD quintiles |  |  |  |  |
| Q1 | 1.25 (1.20-1.30) | 1.23 (1.18-1.28) | 1.33 (1.28-1.39) | 1.13 (1.08-1.17) |
| Q2 | 1.16 (1.11-1.20) | 1.15 (1.11-1.20) | 1.21 (1.17-1.26) | 1.08 (1.04-1.13) |
| Q3 | 1.11 (1.07-1.16) | 1.10 (1.05-1.14) | 1.14 (1.10-1.19) | 1.07 (1.02-1.11) |
| Q4 | 1.05 (1.00-1.09) | 1.04 (1.00-1.08) | 1.06 (1.02-1.11) | 1.02 (0.98-1.06) |
| Q5 | 1.00 (reference) | 1.00 (reference) | 1.00 (reference) | 1.00 (reference) |
| *P for trend* | < 0.001 | < 0.001 | < 0.001 | < 0.001 |
| **HS plus FIB-4 ≥ 1.3** |  |  |  |  |
| RMSSD quintiles |  |  |  |  |
| Q1 | 1.20 (0.98-1.46) | 1.22 (1.00-1.50) | 1.28 (1.05-1.57) | 1.14 (0.93-1.39) |
| Q2 | 1.17 (0.95-1.43) | 1.20 (0.98-1.48) | 1.24 (1.01-1.53) | 1.13 (0.92-1.39) |
| Q3 | 1.16 (0.94-1.43) | 1.19 (0.96-1.46) | 1.21 (0.98-1.49) | 1.14 (0.92-1.41) |
| Q4 | 0.98 (0.78-1.23) | 0.98 (0.78-1.23) | 0.99 (0.79-1.24) | 0.97 (0.78-1.22) |
| Q5 | 1.00 (reference) | 1.00 (reference) | 1.00 (reference) | 1.00 (reference) |
| *P for trend* | 0.022 | 0.010 | 0.002 | 0.093 |
| **HS plus NFS ≥ –1.455** |  |  |  |  |
| RMSSD quintiles |  |  |  |  |
| Q1 |  | 1.27 (1.08-1.51) | 1.42 (1.20-1.68) | 1.23 (1.04-1.45) |
| Q2 |  | 1.13 (0.95-1.34) | 1.22 (1.03-1.45) | 1.11 (0.93-1.32) |
| Q3 |  | 1.17 (0.98-1.40) | 1.23 (1.03-1.46) | 1.16 (0.97-1.38) |
| Q4 |  | 1.05 (0.87-1.26) | 1.07 (0.89-1.29) | 1.04 (0.87-1.25) |
| Q5 |  | 1.00 (reference) | 1.00 (reference) | 1.00 (reference) |
| *P for trend* |  | 0.002 | < 0.001 | 0.010 |

^a^Estimated from parametric proportional hazard models. Multivariable model 1 was adjusted for age, sex, study centre, year of screening exam, smoking, alcohol consumption, physical activity, depression, sleep duration, education level, total energy intake, SBP, and BMI.; model 2: model 1 plus adjustment for HOMA-IR instead of BMI; model 3: model 1 plus adjustment for hs-CRP instead of BMI; and model 4: model 1 plus adjustment for HOMA-IR and hs-CRP.

BMI, Body mass index; CI, Confidence interval; FIB-4, Fibrosis 4 score; HOMA-IR, Homeostasis model assessment of insulin resistance; HR, Hazard ratio; HS, Hepatic steatosis; hsCRP, High-sensitivity C-reactive protein; PY, Person-year; RMSSD, Root mean square of successive differences in RR intervals; SDNN, Standard deviation of normal-to-normal intervals; SBP, Systolic blood pressure

**Supplementary Table 2.** Hazard ratios (95% CI) for the development of hepatic steatosis (HS) or HS with intermediate-to-high probability of advanced fibrosis measured by FIB-4 score, according to heart rate variability, among participants without obesity

| **Quintiles of heart rate variability** | Non-obese participants (n = 140,274) | |
| --- | --- | --- |
|  | Multivariable-adjusted HR^a^ (95% CI) for development of HS | Multivariable-adjusted HR^a^ (95% CI) for development of HS with intermediate-to-high FIB-4 |
| SDNN quintiles |  |  |
| Q1 | 1.30 (1.24-1.36) | 1.25 (1.00-1.56) |
| Q2 | 1.16 (1.11-1.22) | 1.26 (1.01-1.58) |
| Q3 | 1.09 (1.04-1.14) | 1.01 (0.80-1.29) |
| Q4 | 1.02 (0.97-1.07) | 1.07 (0.84-1.36) |
| Q5 | 1.00 (reference) | 1.00 (reference) |
| *P for trend* | < 0.001 | 0.013 |
| RMSSD quintiles |  |  |
| Q1 | 1.41 (1.35-1.48) | 1.39 (1.10-1.75) |
| Q2 | 1.24 (1.19-1.30) | 1.19 (0.94-1.51) |
| Q3 | 1.14 (1.09-1.20) | 1.20 (0.94-1.52) |
| Q4 | 1.08 (1.03-1.14) | 0.94 (0.73-1.22) |
| Q5 | 1.00 (reference) | 1.00 (reference) |
| *P for trend* | < 0.001 | < 0.001 |

^a^Estimated from parametric proportional hazard models. The multivariable model was adjusted for age, sex, study centre, year of screening examination, smoking, alcohol consumption, physical activity, depression, sleep duration, education level, total energy intake, and SBP.

HR, Hazard ratio; CI, Confidence interval; HS, Hepatic steatosis; FIB-4, Fibrosis 4 score; SDNN, Standard deviation of normal-to-normal intervals; RMSSD, Root mean square of successive differences in RR intervals

**Supplementary Table 3.** The incidence of non-alcoholic fatty liver disease (NAFLD) by heart rate variability when NAFLD was defined as a diagnosis of exclusion during follow-up

| **Heart rate variability quintiles** | PY | Incident  cases | Incidence density  (/10^3^ PY) | Age- and sex-adjusted  HR (95% CI) | Multivariable-adjusted HR^a^ (95% CI) | HR (95% CI)^b^  in a model with time-dependent variables |
| --- | --- | --- | --- | --- | --- | --- |
| SDNN |  |  |  |  |  |  |
| Q1 | 115,340 | 6,416 | 55.6 | 1.38 (1.33-1.44) | 1.32 (1.26-1.37) | 1.31 (1.26-1.37) |
| Q2 | 123,039 | 5,594 | 45.5 | 1.22 (1.17-1.27) | 1.20 (1.15-1.25) | 1.20 (1.15-1.25) |
| Q3 | 125,377 | 5,081 | 40.5 | 1.12 (1.08-1.17) | 1.12 (1.08-1.17) | 1.10 (1.06-1.15) |
| Q4 | 125,365 | 4,613 | 36.8 | 1.04 (1.00-1.09) | 1.04 (1.00-1.08) | 1.04 (1.00-1.09) |
| Q5 | 125,368 | 4,327 | 34.5 | 1.00 (reference) | 1.00 (reference) | 1.00 (reference) |
| *P for trend* |  |  |  | < 0.001 | < 0.001 | < 0.001 |
| RMSSD |  |  |  |  |  |  |
| Q1 | 114,083 | 6,795 | 59.6 | 1.50 (1.44-1.57) | 1.42 (1.37-1.48) | 1.44 (1.38-1.51) |
| Q2 | 122,922 | 5,746 | 46.7 | 1.29 (1.24-1.35) | 1.27 (1.22-1.32) | 1.26 (1.20-1.31) |
| Q3 | 125,903 | 5,077 | 40.3 | 1.17 (1.12-1.22) | 1.16 (1.11-1.21) | 1.18 (1.13-1.24) |
| Q4 | 125,806 | 4,511 | 35.9 | 1.08 (1.03-1.13) | 1.08 (1.03-1.13) | 1.08 (1.03-1.13) |
| Q5 | 125,776 | 3,902 | 31.0 | 1.00 (reference) | 1.00 (reference) | 1.00 (reference) |
| *P for trend* |  |  |  | < 0.001 | < 0.001 | < 0.001 |

^a^Estimated from parametric proportional hazard models. The multivariable model was adjusted for age, sex, centre, year of screening examination, smoking, alcohol consumption, physical activity, depressive symptoms, sleep duration, education level, total energy intake, and systolic blood pressure.

^b^Estimated from parametric proportional hazard models with quintiles of each heart rate variability (SDNN and RMSSD), smoking, alcohol consumption, physical activity, depressive symptoms, sleep duration, total energy intake, and systolic blood pressure as time-dependent categorical variables; and baseline age, sex, centre, year of screening exam, and education level as time-fixed variables.

CI, confidence interval; HR, hazard ratio; PY, person-year; RMSSD, root mean square of successive differences in RR intervals; SDNN, standard deviation of normal-to-normal intervals

**Supplementary Table 4.** Hazard ratios (95% CI) for the development of non-alcoholic fatty liver disease (NAFLD) with an intermediate-to-high probability of advanced fibrosis based on fibrosis-4 score and NAFLD fibrosis score by heart rate variability when NAFLD was defined as a diagnosis of exclusion during follow-up

| Heart rate variability quintiles | For development of HS with an intermediate-to-high probability of advanced fibrosis based on fibrosis-4 score | | | For development of HS with an intermediate-to-high probability of advanced fibrosis based on NAFLD fibrosis score | | |  |
| --- | --- | --- | --- | --- | --- | --- | --- |
|  | Incidence density  (/10^3^ PY) | Multivariable-adjusted HR^a^ (95% CI) | HR (95% CI)^b^  in a model with time-dependent variables | Incidence density  (/10^3^ PY) | Multivariable-adjusted HR^a^ (95% CI) | HR (95% CI)^b^  in a model with time-dependent variables | |
| SDNN |  |  |  |  |  |  | |
| Q1 | 2.5 | 1.12 (0.92-1.37) | 1.47 (1.17-1.85) | 4.1 | 1.37 (1.16-1.62) | 1.57 (1.31-1.89) | |
| Q2 | 2.1 | 1.25 (1.02-1.53) | 1.44 (1.13-1.82) | 2.8 | 1.24 (1.05-1.47) | 1.39 (1.15-1.68) | |
| Q3 | 1.6 | 1.12 (0.91-1.38) | 1.41 (1.10-1.80) | 2.2 | 1.13 (0.95-1.35) | 1.27 (1.04-1.54) | |
| Q4 | 1.3 | 1.03 (0.83-1.28) | 1.39 (1.07-1.79) | 1.9 | 1.04 (0.87-1.25) | 1.17 (0.95-1.44) | |
| Q5 | 1.1 | 1.00 (reference) | 1.00 (reference) | 1.6 | 1.00 (reference) | 1.00 (reference) | |
| *P for trend* |  | 0.087 | 0.008 |  | < 0.001 | < 0.001 | |
| RMSSD |  |  |  |  |  |  | |
| Q1 | 2.8 | 1.3 (1.05-1.6) | 1.69 (1.33-2.16) | 4.4 | 1.46 (1.23-1.74) | 1.68 (1.38-2.03) | |
| Q2 | 1.9 | 1.23 (0.99-1.52) | 1.48 (1.15-1.90) | 2.8 | 1.24 (1.04-1.48) | 1.35 (1.11-1.65) | |
| Q3 | 1.7 | 1.26 (1.01-1.56) | 1.41 (1.09-1.84) | 2.4 | 1.28 (1.07-1.53) | 1.26 (1.02-1.55) | |
| Q4 | 1.2 | 1.02 (0.81-1.29) | 1.37 (1.04-1.80) | 1.8 | 1.09 (0.90-1.32) | 1.23 (0.99-1.53) | |
| Q5 | 1.0 | 1.00 (reference) | 1.00 (reference) | 1.4 | 1.00 (reference) | 1.00 (reference) | |
| *P for trend* |  | 0.004 | < 0.001 |  | < 0.001 | < 0.001 | |

^a^Estimated from parametric proportional hazard models. The multivariable model was adjusted for age, sex, study centre, year of screening examination, smoking, alcohol consumption, physical activity, depressive symptoms, sleep duration, education level, total energy intake, and systolic blood pressure.

^b^Estimated from parametric proportional hazard models with quintiles of each heart rate variability (SDNN, RMSSD), smoking, alcohol consumption, physical activity, depressive symptoms, sleep duration, total energy intake, and systolic blood pressure as time-dependent categorical variables; and baseline age, sex, centre, year of screening exam, and education level as time-fixed variables.

CI, confidence interval; HR, hazard ratio; HS, hepatic steatosis; PY, person-year; RMSSD, root mean square of successive differences in RR intervals; SDNN, standard deviation of normal-to-normal intervals

**Supplementary Table 5.** Incidence of high Hepatic Steatosis Index (>36) heart rate variability

| **Heart rate variability quintiles** | PY | Incident  cases | Incidence density  (/10^3^ PY) | Age sex adjusted  HR (95% CI) | Multivariable-adjusted HR^a^ (95% CI) | | HR (95% CI)^b^  in a model with time-dependent variables |
| --- | --- | --- | --- | --- | --- | --- | --- |
|  |  |  |  |  | Model 1 | Model 2 |  |
| SDNN |  |  |  |  |  |  |  |
| Q1 | 109,751 | 3,042 | 27.7 | 1.33 (1.26-1.40) | 1.30 (1.23-1.37) | 1.27 (1.19-1.34) | 1.36 (1.30-1.43) |
| Q2 | 118,321 | 2,898 | 24.5 | 1.19 (1.13-1.26) | 1.19 (1.13-1.26) | 1.17 (1.11-1.24) | 1.22 (1.17-1.28) |
| Q3 | 121,046 | 2,705 | 22.3 | 1.09 (1.03-1.15) | 1.10 (1.04-1.16) | 1.09 (1.03-1.15) | 1.14 (1.08-1.19) |
| Q4 | 121,320 | 2,637 | 21.7 | 1.05 (0.99-1.11) | 1.05 (1.00-1.11) | 1.05 (0.99-1.11) | 1.07 (1.01-1.12) |
| Q5 | 121,699 | 2,554 | 21.0 | 1.00 (reference) | 1.00 (reference) | 1.00 (reference) | 1.00 (reference) |
| *P for trend* |  |  |  | < 0.001 | < 0.001 | < 0.001 | < 0.001 |
| RMSSD |  |  |  |  |  |  |  |
| Q1 | 108,262 | 3,210 | 29.7 | 1.41 (1.33-1.49) | 1.37 (1.29-1.44) | 1.37 (1.29-1.46) | 1.40 (1.33-1.47) |
| Q2 | 117,549 | 2,944 | 25.0 | 1.24 (1.17-1.31) | 1.24 (1.17-1.31) | 1.24 (1.17-1.32) | 1.31 (1.24-1.37) |
| Q3 | 122,031 | 2,695 | 22.1 | 1.10 (1.04-1.16) | 1.11 (1.05-1.17) | 1.11 (1.05-1.17) | 1.15 (1.09-1.21) |
| Q4 | 121,996 | 2,612 | 21.4 | 1.07 (1.02-1.14) | 1.09 (1.03-1.15) | 1.09 (1.03-1.15) | 1.06 (1.01-1.12) |
| Q5 | 122,300 | 2,375 | 19.4 | 1.00 (reference) | 1.00 (reference) | 1.00 (reference) | 1.00 (reference) |
| *P for trend* |  |  |  | < 0.001 | < 0.001 | < 0.001 | < 0.001 |

^a^Estimated from parametric proportional hazard models. The multivariable model was adjusted for age, sex, centre, year of screening examination, smoking, alcohol consumption, physical activity, depressive symptoms, sleep duration, education level, total energy intake, and systolic blood pressure.

^b^Estimated from parametric proportional hazard models with quintiles of each heart rate variability (SDNN and RMSSD), smoking, alcohol consumption, physical activity, depressive symptoms, sleep duration, total energy intake, and systolic blood pressure as time-dependent categorical variables; and baseline age, sex, centre, year of screening exam, and education level as time-fixed variables.

CI, confidence interval; HR, hazard ratio; PY, person-year; RMSSD, root mean square of successive differences in RR intervals; SDNN, standard deviation of normal-to-normal intervals

**Supplementary Table 6.** Hazard ratio (95% CI) of hepatic steatosis by other covariates

|  | Crude HR (95% CI) | Multivariate-adjusted HR (95% CI) |
| --- | --- | --- |
| Age per 5-yr increment | 1.15 (1.14-1.16) | 1.12 (1.11-1.13) |
| SBP per 5 mmHg increment | 1.19 (1.18-1.19) | 1.09 (1.09-1.10) |
| Sex |  |  |
| Female | 1.00 (reference) | 1.00 (reference) |
| Male | 3.26 (3.18-3.34) | 2.60 (2.51-2.69) |
| Alcohol intake |  |  |
| 0 g/day | 1.00 (reference) | 1.00 (reference) |
| <10 g/day | 1.13 (1.08-1.17) | 0.93 (0.90-0.97) |
| 10-19.9 g/day | 1.80 (1.73-1.88) | 0.98 (0.93-1.02) |
| ≥20 g/day | 2.43 (2.31-2.56) | 0.93 (0.88-0.98) |
| Smoking |  | — |
| Never | 1.00 (reference) | 1.00 (reference) |
| Ever | 1.83 (1.78-1.89) | 0.98 (0.95-1.01) |
| Current | 2.58 (2.50-2.66) | 1.23 (1.19-2.28) |
| Physical activity |  |  |
| Inactive | 1.00 (reference) | 1.00 (reference) |
| Minimal | 1.18 (1.14-1.21) | 1.00 (0.98-1.03) |
| HEPA | 1.13 (1.09-1.17) | 0.98 (0.94-1.01) |
| Total energy intake |  |  |
| Q1 | 1.00 (reference) | 1.00 (reference) |
| Q2 | 1.06 (1.00-1.12) | 0.97 (0.92-1.03) |
| Q3 | 1.18 (1.11-1.25) | 1.00 (0.94-1.06) |
| Q4 | 1.21 (1.14-1.28) | 0.99 (0.93-1.04) |
| Q5 | 1.34 (1.27-1.42) | 1.03 (0.98-1.09) |
| Educational level |  |  |
| <12 years | 1.00 (reference) | 1.00 (reference) |
| ≥ 12 years | 1.07 (1.03-1.11) | 0.90 (0.87-0.94) |
| Sleep duration |  |  |
| ≤6 hours | 1.00 (reference) | 1.00 (reference) |
| >6-7 hours | 0.96 (0.93-1.00) | 0.91 (0.87-0.94) |
| >7-8 hours | 0.79 (0.76-0.82) | 0.84 (0.80-0.87) |
| >8-9 hours | 0.61 (0.58-0.64) | 0.82 (0.78-0.86) |
| ≥ 9 hours | 0.49 (0.44-0.53) | 0.80 (0.73-0.87) |
| CESD-score |  |  |
| <8 | 1.00 (reference) | 1.00 (reference) |
| 8-15 | 0.96 (0.93-0.99) | 1.03 (1.00-1.06) |
| 16-24 | 0.82 (0.78-0.86) | 1.04 (0.99-1.09) |
| ≥25 | 0.74 (0.69-0.81) | 1.06 (0.98-1.14) |

^a^Estimated from parametric proportional hazard models. The multivariate models included heart rate variability (SDNN and RMSSD), centre, year of screening examination, and all other variables listed for the model.

CI, confidence interval

Supplementary Table 7. Baseline characteristics of study participants by SDNN quintiles

| Characteristics | SDNN quintiles | | | | | *p* for trend |
| --- | --- | --- | --- | --- | --- | --- |
|  | Q1 | Q2 | Q3 | Q4 | Q5 |  |
| Number | 23,943 | 30,751 | 31,054 | 31,227 | 31,311 |  |
| Age (years)^a^ | 39.2 (7.4) | 37.3 (6.4) | 36.1 (6.0) | 35.1 (5.7) | 33.9 (5.5) | < 0.001 |
| Men (%) | 41.6 | 37.4 | 36.2 | 35.9 | 36.3 | < 0.001 |
| Current smoker (%) | 15.2 | 14.2 | 13.2 | 13.5 | 13.4 | < 0.001 |
| Alcohol intake (%)^c^ | 27.2 | 24.8 | 24.2 | 24.2 | 24.2 | < 0.001 |
| HEPA (%) | 13.3 | 13.2 | 14.0 | 15.3 | 16.5 | < 0.001 |
| High education (%)^d^ | 87.9 | 86.8 | 88.9 | 88.6 | 86.0 | < 0.001 |
| Depression (%)^e^ | 11.6 | 11.4 | 12.0 | 12.4 | 12.1 | < 0.001 |
| Sleep duration (hours)^a^ | 6.59 (1.13) | 6.63 (1.14) | 6.63 (1.14) | 6.64 (1.14) | 6.64 (1.16) | < 0.001 |
| Systolic BP (mmHg)^a^ | 107.4 (12.2) | 105.1 (11.4) | 104.2 (11.1) | 103.7 (11.0) | 103.1 (11.0) | < 0.001 |
| Body mass index (kg/m^2^) | 22.3 (2.7) | 21.9 (2.6) | 21.7 (2.6) | 21.6 (2.6) | 21.5 (2.5) | < 0.001 |
| HOMA-IR^c^ | 1.09 (0.74-1.54) | 1.02 (0.69-1.45) | 1.01 (0.68-1.44) | 1.00 (0.68-1.42) | 0.96 (0.66-1.37) | < 0.001 |
| hsCRP (mg/L)^c^ | 0.4 (0.2-0.8) | 0.3 (0.2-0.6) | 0.3 (0.2-0.6) | 0.3 (0.2-0.6) | 0.3 (0.2-0.6) | < 0.001 |
| Total energy intake^b,f^ | 1494.4 (1139.6-1877.2) | 1487.2 (1132.6-1868.6) | 1477.9 (1124.9-1859.5) | 1479.7 (1114.6-1876.7) | 1470.4 (1107.0-1870.0) | < 0.001 |

Data are expressed as ^a^mean (standard deviation), ^b^median (interquartile range), or percentage.

^c^≥20 g/day; ^d^≥college graduate; ^e^≥16 of CES-D score; ^f^among 108,475 participants with plausible estimated energy intake levels (within three standard deviations from the log-transformed mean energy intake)

Supplementary Table 8. Baseline characteristics of study participants by RMSSD quintiles

| Characteristics | RMSSD quintiles | | | | | P for trend |
| --- | --- | --- | --- | --- | --- | --- |
|  | Q1 | Q2 | Q3 | Q4 | Q5 |  |
| Number | 29,712 | 30,800 | 31,086 | 31,272 | 31,416 |  |
| Age (years)^a^ | 39.2 (7.4) | 37.3 (6.5) | 36.2 (6.1) | 35.1 (5.7) | 33.8 (5.4) | < 0.001 |
| Men (%) | 45.4 | 39.1 | 36.4 | 35.0 | 31.8 | < 0.001 |
| Current smoker (%) | 15.8 | 14.7 | 13.4 | 13.1 | 12.5 | < 0.001 |
| Alcohol intake (%)^c^ | 28.4 | 25.5 | 24.2 | 23.7 | 23.0 | < 0.001 |
| HEPA (%) | 12.8 | 13.2 | 13.9 | 15.1 | 17.2 | < 0.001 |
| High education (%)^d^ | 84.3 | 85.6 | 85.6 | 86.8 | 85.6 | < 0.001 |
| Depression (%)^e^ | 11.0 | 11.3 | 11.9 | 12.6 | 13.9 | < 0.001 |
| Sleep duration (hours)^a^ | 6.58 (1.12) | 6.62 (1.13) | 6.64 (1.14) | 6.64 (1.15) | 6.65 (1.17) | < 0.001 |
| Systolic BP (mmHg)^a^ | 108.5 (12.3) | 105.4 (11.3) | 104.2 (11.0) | 103.3 (10.8) | 102.2 (10.6) | < 0.001 |
| Body mass index (kg/m^2^) | 22.4 (2.7) | 22.0 (2.6) | 21.7 (2.6) | 21.5 (2.5) | 21.4 (2.5) | < 0.001 |
| HOMA-IR^c^ | 1.12 (0.75-1.59) | 1.04 (0.71-1.47) | 1.01 (0.68-1.43) | 0.98 (0.67-1.40) | 0.94 (0.64-1.35) | < 0.001 |
| hsCRP (mg/L)^c^ | 0.4 (0.2-0.8) | 0.3 (0.2-0.7) | 0.3 (0.2-0.6) | 0.3 (0.2-0.6) | 0.3 (0.2-0.5) | < 0.001 |
| Total energy intake^b,f^ | 1502.1 (1150.4-1880.2) | 1487.3 (1139.1-1871.1) | 1480.8 (1120.5-1868) | 1477.9 (1116.9-1877.2) | 1459.5 (1093.3-1858.9) | < 0.001 |

Data are expressed as ^a^mean (standard deviation), ^b^median (interquartile range), or percentage.

^c^≥10 g/day; ^d^≥ college graduate; ^e^≥16 of CES-D score; ^f^among 108,475 participants with plausible estimated energy intake levels (within three standard deviations from the log-transformed mean energy intake)
